# Coronary lesion complexity in patients with heterozygous familial hypercholesterolemia hospitalized for acute myocardial infarction: data from the RICO survey

**DOI:** 10.1186/s12944-021-01467-z

**Published:** 2021-05-04

**Authors:** Hermann Yao, Michel Farnier, Laura Tribouillard, Frédéric Chague, Philippe Brunel, Maud Maza, Damien Brunet, Luc Rochette, Florence Bichat, Yves Cottin, Marianne Zeller

**Affiliations:** 1 Cardiology Department, University Hospital Center Dijon Bourgogne, Dijon, France; 2 PEC2, EA 7460, UFR Health Sciences, University of Bourgogne Franche Comté, Dijon, France; 3 Private Hospital Dijon Bourgogne, Dijon, France

**Keywords:** Familial hypercholesterolemia, Myocardial infarction, Complex coronary lesions, LDL cholesterol

## Abstract

**Background:**

Although patients with familial heterozygous hypercholesterolemia (FH) have a high risk of early myocardial infarction (MI), the coronary artery disease (CAD) burden in FH patients with acute MI remains to be investigated.

**Methods:**

The data for all consecutive patients hospitalized in 2012–2019 for an acute MI and who underwent coronary angiography were collected from a multicenter database (RICO database). FH (*n* = 120) was diagnosed using Dutch Lipid Clinic Network criteria (score ≥ 6). We compared the angiographic features of MI patients with and without FH (score 0–2) (*n* = 234) after matching for age, sex, and diabetes (1:2).

**Results:**

Although LDL-cholesterol was high (208 [174–239] mg/dl), less than half of FH patients had chronic statin treatment. When compared with non-FH patients, FH increased the extent of CAD (as assessed by SYNTAX score; *P* = 0.005), and was associated with more frequent multivessel disease (*P* = 0.004), multiple complex lesions (*P* = 0.022) and significant stenosis location on left circumflex and right coronary arteries. Moreover, FH patients had more multiple lesions, with an increased rate of bifurcation lesions or calcifications (*P* = 0.021 and *P* = 0.036, respectively). In multivariate analysis, LDL-cholesterol levels (OR 1.948; 95% CI 1.090–3.480, *P* = 0.024) remained an independent estimator of anatomical complexity of coronary lesions, in addition to age (OR 1.035; 95% CI 1.014–1.057, *P* = 0.001).

**Conclusions:**

FH patients with acute MI had more severe CAD, characterized by complex anatomical features that are mainly dependent on the LDL-cholesterol burden. Our findings reinforce the need for more aggressive preventive strategies in these high-risk patients, and for intensive lipid-lowering therapy as secondary prevention.

## Introduction

Heterozygous familial hypercholesterolemia (FH) is one of the most common autosomal dominant genetic diseases [1], with an estimated prevalence of 1/250 in Western countries. It is characterized by high levels of LDL cholesterol (LDL-C) [2, 3], resulting in most cases from a mutation of the LDL receptor (LDL-R), apolipoprotein B (apoB), or proprotein convertase subtilisin/kexin type 9 (PCSK9). The most commonly used routine diagnostic criteria are the Dutch Lipid Clinic Network (DLCN) criteria, based primarily on elevated LDL-C levels and the presence of a family and personal history of premature coronary heart disease [4]. In uncertain cases, a genetic analysis can be used to confirm the diagnosis and to provide sensitive and specific molecular family screening.

Patients with FH present a very high cardiovascular (CV) risk and are therefore exposed to the occurrence of coronary events at an early age [5, 6]. On average, patients with FH have a risk of early coronary artery disease (CAD) that is 13 times higher than in the general population [5]. When individuals do not respond to treatment, fatal or non-fatal coronary events occur in approximately 50% of men < 50 y and 30% of women < 60 y [6]. FH is often found after an individual has a myocardial infarction (MI), with an estimated prevalence between 1.6 and 4.3% [7, 8]. Furthermore, FH patients have an unfavorable prognosis after MI, with a risk of recurrence of cardiovascular or coronary events that is 2 to 3 times higher than the average [9, 10]. However, there are wide variations in the extent of CAD and in the level of coronary calcifications between individuals with genetically determined FH, suggesting the need for a better understanding of its specificities [4, 11].

Thus, while the clinical course of these patients is relatively well known, there is a paucity of research focused on the associated coronary lesions. Although CAD is more frequently associated with multi-vessel disease in FH patients, there are significant variations in prevalence [12–14]. Using the Gensini angiographic score, Wang [8] and Li [15] showed that CAD was more severe in patients with FH than in those without FH (according to the DLCN criteria). Findings from a series of 104 asymptomatic age-matched patients found that coronary lesions in CAD patients with genetically confirmed heterozygous FH are more diffuse and calcified than in patients without a genetic mutation [16]. The angiographic characteristics of the anatomical complexity of coronary lesions, such as number, size, lesion length, and multiple lesions are risk factors that worsen prognosis after MI [17, 18]. In addition, targeted therapeutic strategies appear to be more beneficial in patients with complex coronary anatomy [19]. However, the complexity of coronary lesions in symptomatic patients with FH has not yet been described.

The objective of this study was therefore to characterize the severity and complexity of coronary lesions on coronary angiography in FH patients hospitalized for acute MI.

## Patients and methods

### Study population, selection criteria

This retrospective study was conducted using data from the RICO (Côte d’Or Myocardial Infarction Observatory) database [20]. RICO is an ongoing survey that has included all consecutive patients aged at least 18 years hospitalized for an acute MI in a coronary care unit of all public or privately funded hospitals receiving MI emergencies in the region of Côte d’Or (France) since 2001. Cases were ascertained by the prospective collection of consecutive admissions. MI was identified by an increase in serum troponin I (greater than the upper limit of normal for each hospital) and clinical symptoms of ischemia and/or characteristic electrocardiographic signs.

For the current study, patients hospitalized for an acute MI at the Dijon University Hospital and who underwent coronary angiography between 2012 and 2019 were included. A retrospective analysis of coronary angiographies was performed using a digital medium (Intellispace Cardiovascular™). Acute MI was defined according to the current universal definition [21].

The probability of FH was calculated from the sum of the points from an adapted version of the Dutch Lipid Clinic Network (DLCN) score criteria [4]: family history of premature CAD in a first-degree relative (male < 55 years and female < 60 years; 1 point); personal history of premature CAD (2 points) or vascular disease (1 point); and LDL-cholesterol (LDL-C) value: 330 mg/dL [8 points], 250–329 mg/dL [5 points], 190-249 mg/dL [3 points], 155–189 mg/dL [1 point]). In individuals on lipid-lowering therapy, LDL-C at admission was corrected for the drug class: statins (130%), ezetimibe (120%), and statins and ezetimibe (140%). A conservative correction factor for statin treatment (130%) was chosen because moderate intensity statins are mostly used in France. The presence of tendon xanthomas or corneal arches and a family history of hypercholesterolemia or vascular disease were not recorded in the database. Missing information was counted as zero. For each patient, the diagnosis of FH was considered certain or probable when the total score was ≥6, and absent when the score was < 3.

**Fig. 1:**
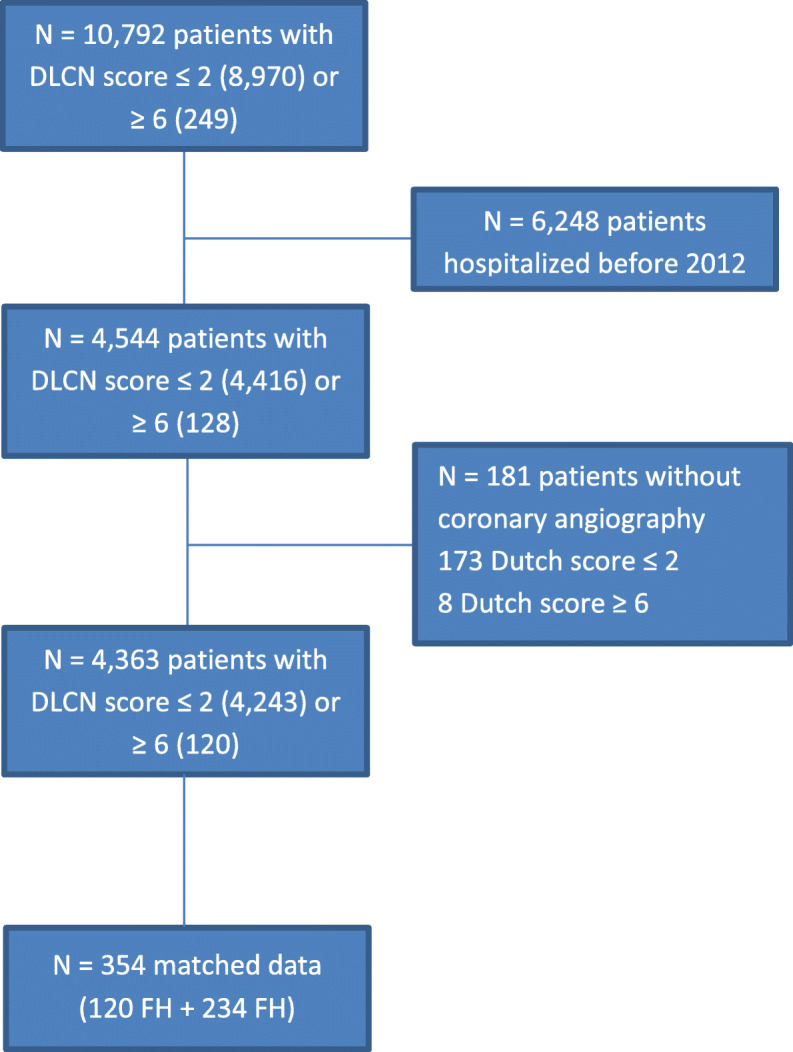
Flow chart

Among the patients included in the RICO database, 120 were categorized as certain or probable FH (score ≥ 6) and 4243 as unlikely FH (non-FH; score < 3). The characteristics of the main cohort have already been described [7]. The 120 FH patients were matched (1:2) with 234 non-FH patients from the database based on age (± standard deviation), sex, and presence of type 2 diabetes. The flow chart is described in Fig. 1.

We analyzed risk factors, CV history (defined as history of MI, percutaneous coronary intervention (PCI) or coronary artery bypass graft surgery (CABG), lipid-lowering medications, time to admission, clinical data at admission, and hospital complications. Left ventricular ejection fraction (LVEF) was assessed within 12 h of admission using the Simpson biplane method. Blood lipids and other biological parameters were obtained on admission, except for peak troponin Ic, which was determined from 3 samples taken at 8-h intervals in the first 24 h after admission. We also calculated the GRACE score for each patient [22] according to age, Killip class, systolic blood pressure, heart rate, ST segment changes, cardiac arrest on admission, creatinine levels and cardiac enzyme elevation.

### Evaluation of coronary angiography lesions and patient management

Coronary angiography images were reviewed by two trained interventional cardiologists who were blinded to the patient’s group. There was a discrepancy in 7 cases, which were then adjudicated through a joint review.

Coronary angiography was considered normal when the angiographic images did not show any visible atheromatous plaque or spastic phenomena. Coronary lesions were considered non-significant for stenoses < 50% (and significant when stenoses were ≥ 50%). Depending on the number of diseased vessels (≥ 2.5 mm), multivessel CAD was considered when they were located on the left anterior descending artery and/or the diagonal branches, the left circumflex artery and marginal arteries, the right coronary artery (and/or the posterior interventricular or the left retro ventricular arteries), with or without involvement of the left main artery.

For each patient, initial SYNTAX scores (before the revascularization procedure) and residual SYNTAX scores (after the revascularization procedure) were calculated [23].

Complex lesions were identified according to pre-specified criteria from the CHAMPION-PHOENIX [24] and DAPT [18] studies: left main lesion, long lesion > 20 mm, multiple lesions (> 2 lesions per vessel), bifurcation lesion (with side branch > 1.5 mm), significant tortuosity (two between 45 and 90° or one greater than 90° in the vicinity of the lesion), thrombus, angulation, eccentricity, and stenting of a saphenous graft. Moderate calcifications (radio-opaque density during the cardiac cycle and affecting only on one edge of the vascular wall) or severe calcifications (radio-opaque density visualized even in the absence of cardiac movement before injection of the contrast agent and most often throughout the arterial wall) were identified. Multiple complex lesions were defined by the presence of several complex lesions [17].

Coronary angiographic data were collected: TIMI flow, culprit artery, number of diseased vessels, stents (number, diameter, length and type), and revascularization strategies (thrombectomy, PCI and CABG). In-hospital CV events were also analyzed (recurrent MI, stroke, heart failure, or death). Heart failure (HF) was defined by Killip class > 1.

### Ethics approval and consent to participate

Informed consent was obtained for each patient prior to inclusion in the study. The study protocol was authorized by the Ethics Committee of the Dijon University Hospital.

### Statistical analysis

The categorical variables, expressed in numbers and percentages, were compared using Pearson’s Chi-square tests or Fisher’s exact tests. Continuous variables, presented as medians [interquartile range], were compared by the Student or Mann-Whitney/Wilcoxon test. The normality of the variables was determined using Kolmogorov-Smirnov test. Significance was set at *P* < 0.05.

Multivariate logistic regression analyses were used to identify factors associated with multiple complex coronary lesions (> 1 complex anatomical feature) or multi-vessel CAD (> 1 coronary vessel with significant stenosis). Multivariate models were built by including predictive variables in univariate analysis, with an inclusion threshold of *P* < 0.10. Although not significant in univariate analyses, sex and diabetes were included as forced variables in the multivariate models, given their impact on the dependent variable. The threshold for defining high CRP levels (CRP > 3 mg/L) was chosen for its clinical relevance. Statistical analyses were performed using SPSS version 12.0.1 (IBM Inc.).

## Results

FH patients (DLCN score ≥ 6) (*n* = 120) were compared to non-FH patients (DLCN < 3) (*n* = 234) (Table 1). FH patients had a higher incidence of hypertension (*P* = 0.002) and, as expected, a higher incidence of personal or family history of CAD (*p* < 0.001). Statins (*P* < 0.001) and ezetimibe (*P* < 0.001) were prescribed more often to FH patients. However, although LDL-cholesterol was high (208 [174–239] mg/dL), less than half of FH patients had a prescription for chronic statin treatment. As expected, FH patients had higher levels of LDL-C and triglycerides (*P* < 0.001 for both). On admission, the rate of ST-segment-elevation MI was similar for both groups (*P* = 0.355), as was the GRACE risk score (*P* = 0.20).

**Table 1:** Baseline characteristics. (n (%) or median (IQR))

	Dutch Lipid Clinic Network score 0–2 ***N*** = 234	Dutch Lipid Clinic Network score ≥ 6 ***N*** = 120	***P***
**CV risk factors**			
Age, *years*	52 (46–59)	51 (46–59)	0.925
Female	89 (38%)	43 (36%)	0.685
BMI, *kg/m*^*2*^	27 (23–30)	27 (24–31)	0.098
Hypercholesterolemia	61 (26%)	86 (72%)	< 0.001
Hypertension	87 (37%)	65 (54%)	0.002
Diabetes	45 (19%)	17 (14%)	0.235
Smoking	137 (59%)	68 (57%)	0.734
Prior CAD	17 (7%)	25 (21%)	< 0.001
Family history of CAD	13 (6%)	87 (73%)	< 0.001
Stroke	14 (6%)	7 (6%)	0.955
PAD	6 (3%)	6 (5%)	0.232
**Medications on admission**			
Ezetrol	3 (1%)	14 (12%)	< 0.001
Fibrate	8 (3%)	1 (1%)	0.283
Statins	31 (13%)	56 (47%)	< 0.001
**Discharge medications**			
Ezetrol	4 (2%)	12 (10%)	< 0.001
Fibrate	2 (1%)	0 (0%)	0.551
Statins	212 (91%)	111 (93%)	0.549
**Clinical data**			
HR, *beats/min*	77 [66–90]; *n* = 228	80 [70–94]; n = 118	0.197
SBP, *mmHg*	139 ± 29; *n =* 228	145 ± 26; *n* = 118	0.048
DBP, *mmHg*	85 ± 20; *n =* 228	90 ± 19; *n* = 117	0.033
Time to admission, *min*	171 [97–388]; *n* = 227	175 [93–429]; *n* = 113	0.964
LVEF, *%*	55 [45–60]; *n* = 233	55 [45–60]	0.617
LVEF < 40%	26 (11%)	7 (6%)	0.104
GRACE Score	116 [96–138]; *n* = 224	110 [93–131]; *n* = 115	0.200
HF	37 (16%)	17 (14%)	0.684
STEMI	133 (57%)	62 (52%)	0.355
Anterior wall location	86 (37%)	35 (29%)	0.154
**Biological data**			
Total cholesterol, m*g/dL*	194 [169–214]	285 [250–320]	< 0.001
HDL cholesterol. m*g/dL*	47 [3658]	45 [36–54]	0.194
LDL cholesterol. m*g/dL*	119 [95–138]	208 [174–239]	< 0.001
LDL cholesterol, corrected ≥190 mg/dL	0 (0%)	117 (98%)	< 0.001
Triglycerides. mg/d*L*	125 [85–176]	149 [103–221]	0.001
CRP ≥ 3 mg/L	130 (56%)	82 (68%)	0.020

Data are expressed as n (%) or medians (IQR)

*CRP* C-reactive protein, *PAD* peripheral artery disease, *BMI* Body Mass index, *CAD* coronary artery disease, *HF* Herat failure, *HR* Heart rate, *SBP* systolic blood pressure, *DBP* Diastolic blood pressure, *LVEF* Left ventricular ejection fraction, *STEMI* ST segment elevation MI

The median length of stay in the coronary care unit was 4 [3–5] days for both groups. FH and no-FH patients had similar rates of in-hospital events (HF: 20 (16.7%) vs 41 (17.5%), *P* = 0.840; recurrent MI: 2 (1.7%) vs 3 (1.3%), *P* = 1; stroke: 1 (0.8%) vs 1 (0.4%), *P* = 1; death 1 (0.8%) vs 2 (0.9%), *P* = 1).

**Fig. 2:**
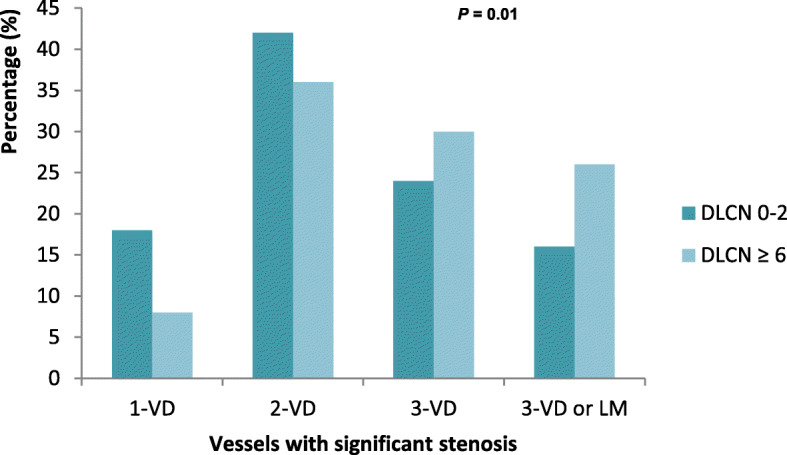
Rate of vessels with significant stenosis

Angiographic data are shown in Table 2. The percentage of optically healthy coronary arteries was much less frequent in FH patients than in non-FH patients, 3% vs 10% (*P* = 0.029), respectively, and 4 FH patients had coronary arteries without stenosis. Compared to the non-FH group, patients in the FH group had a higher initial SYNTAX score (11 [6–20] vs 8 [3–15], *P* = 0.005) and more frequent multivessel disease (56% versus 40%, *P* = 0.01) (Fig. 2). In contrast, the residual SYNTAX score was comparable between the two groups (*P* = 0.47). In FH patients, significant lesions were more often located on left circumflex and marginal arteries (*p* = 0.028), right coronary (*P* = 0.041) and the left retro ventricular artery (*P* = 0.04). On the other hand, no difference was found for the location of the culprit artery (*P* = 0.213). The rate of PCI (*P* = 0.84) and the number of implanted stents (*P* = 0.96) were not significantly different between groups, but CABG was more common in FH patients (*P* = 0.037).

**Table 2:** Coronary angiography data and revascularization procedures

	Dutch Lipid Clinic Network score 0–2 ***N*** = 234	Dutch Lipid Clinic Network score ≥ 6 ***N*** = 120	***P***
SYNTAX Score (initial)	8 [3–15]; *n* = 225	11 [6–20]; *n* = 119	0.005
SYNTAX score (residual)	2 (0–7); *n* = 171	2 (0–8); *n* = 85	0.472
Optically normal arteries	23 (10%)	4 (3%)	0.029
Significant stenosis			
Left main	6 (3%)	5 (4%)	0.519
LAD	120 (51%)	73 (61%)	0.088
Diagonal branch	41 (18%)	30 (25%)	0.096
LAD or diagonal branch	132 (56%)	79 (66%)	0.087
Cx	64 (27%)	39 (33%)	0.313
Marginal artery	29 (12%)	30 (25%)	0.003
Cx or marginal artery	81 (35%)	56 (47%)	0.028
RCA	98 (42%)	64 (53%)	0.041
PIA	9 (4%)	6 (5%)	0.610
LRA	9 (4%)	11 (9%)	0.040
RCA or PIA or LRA	107 (46%)	70 (58%)	0.025
Multi-vessel disease	93 (40%)	67 (56%)	0.004
Culprit artery	*N* = 188	*N* = 106	0.213
Left main	2 (1%)	4 (4%)	
LAD	83 (44%)	48 (45%)	
Cx	29 (16%)	21 (20%)	
RCA	74 (39%)	33 (31%°	
TIMI flow < 2 on culprit artery	92/188 (49%)	56/106 (53%)	0.521
**Revascularisation**			
PCI	172 (74%)	87 (73%)	0.840
Thrombectomy	62/173 (36%)	30/91 (33%)	0.642
CABG	11/223 (5%)	13/118 (11%)	0.037
**Stent number**	*N* = 174	*N* = 88	0.969
0	15 (9%)	6 (7%)	
1	129 (74%)	66 (75%)	
2	25 (14%)	13 (15%)	
3	5 (3%)	3 (3%)	
**Stent type**	*N* = 159	*N* = 82	0.312
BMS	36 (23%)	14 (17%)	
DES	123 (77%)	68 (83%)	
**Stent diameter > 3 mm**	100/159 (63%)	46/82 (56%)	0.279

Data are expressed as n (%) or medians (IQR)

*LAD* left anterior descending, *RCA* right coronary artery, *Cx* left circumflex, *CABG* coronary artery bypass graft, *BMS* bare metal stent, *DES* drug-eluting stent, *PCI* percutaneous coronary intervention, *TIMI* Thrombolysis in acute myocardial infarction, *PIA* posterior interventricular artery, *LRA* left retroventricular artery

**Fig. 3:**
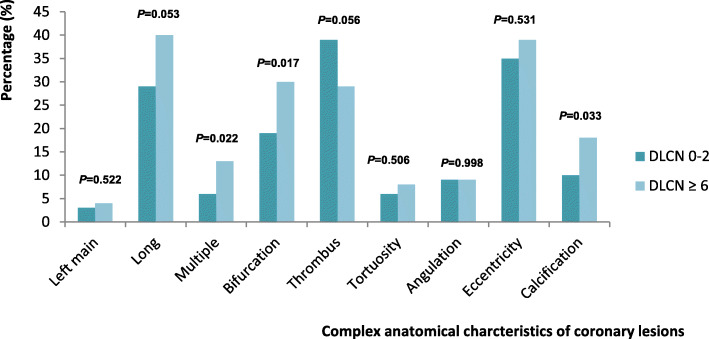
Complex anatomical characteristics of coronary lesions

The number of coronary lesions and their complexity characteristics are reported in Table 3 and Fig. 3. There was no difference between the 2 groups on the overall distribution of the number of complex anatomical features (*P* = 0.129). However, there was a non-significant trend towards more multiple complex lesions (> 1) in FH patients (*P* = 0.053). Our findings indicate that FH patients had more multiple lesions (*P* = 0.022), bifurcation lesions (*P* = 0.017), and calcified lesions (*P* = 0.033) (Fig. 3). Finally, there was a trend in toward longer lesions FH patients (*P* = 0.053), but with less thrombotic burden (*P* = 0.056).

**Table 3:** Anatomical complexity of the coronary lesions

	Dutch Lipid Clinic Network score 0–2 ***N*** = 234	Dutch Lipid Clinic Network score ≥ 6 ***N*** = 120	***P***
**Number of complex characteristics**			0.129
0	56 (24%)	23 (19%)	
1	65 (28%)	26 (22%)	
2	73 (31%)	37 (31%)	
3	25 (11%)	18 (15%)	
4	10 (4%)	11 (9%)	
5	4 (2%)	3 (2%)	
6	0 (0%)	2 (2%)	
7	1 (0.4%)	0 (0%)	
**Multiple complex lesions (number > 1)**	113 (48%)	71 (59%)	0.053

Data are expressed as n (%)

In multivariate analysis, only age (OR 1.033; 95% CI 1.011–1.055) and LDL-cholesterol level (OR 2.141; 95% CI 1.161–3.949) were associated with lesion complexity (> 1 complex anatomical feature) after adjustment for gender, diabetes, chronic statin therapy, FH diagnosis, and a CRP ≥ 3 mg/L (Table 4). The presence of FH, which tended to be associated with multiple complex lesions in univariate analysis, did not persist after adjustment for LDL-C. Furthermore, given the close link between inflammation and hypercholesterolemia, we tested the interaction between CRP and LDL-cholesterol in the multivariate model (*P* interaction = 0.005). The introduction of this interaction did not alter the conclusions of the model. Table 5 shows the variables associated with multivessel disease. Neither FH nor LDL-C levels persist as predictors when adjusted for confounding factors. However, high CRP levels were strongly associated with the development of multivessel disease, as was age (*P* = 0.004 and *P* = 0.002, respectively).

**Table 4:** Logistic regression analysis to estimate lesion anatomical complexity (> 1 complex lesion)

	Univariate		Multivariate	
Variable	OR (95% CI)	*P*	OR (95% CI)	*P*
Female (vs male)	0.800 (0.520–1.232)	0.311	0.570 (0.346–0.940)	0.028
Age, per y	1.027 (1.009–1.045)	0.003	1.035 (1.014–1.057)	0.001
Diabetes (vs no diabetes)	1.150 (0.663–1.993)	0.620	0.889 (0.481–1.642)	0.707
Prior CAD (vs no CAD)	1.584 (0.818–3.068)	0.173	–	
Chronic statins (vs no statins)	0.822 (0.506–1.334)	0.427	–	
FH (DLCN score ≥ 6 vs ≤ 2)	1.246 (0.997–1.556)	0.053	0.890 (0.628–1.259)	0.510
LDL cholesterol. *Per g/L*	1.759 (1.215–2.546)	0.003	1.948 (1.090–3.480)	0.024
CRP ≥ 3 mg/L (vs CRP < 3 mg/L)	1.590 (1.036–2.438)	0.034	1.366 (0.873–2.136)	0.172

*OR* Odds ratio, *CI* confidence interval, *FH* familial hypercholesterolemia, *DLCN* Dutch Lipid Clinic Network, *LDL* Low density lipoprotein, *CAD* coronary artery disease, *CRP* C-Reactive Protein

**Table 5:** Logistic regression analysis to estimate multivessel disease

	Univariate		Multivariate	
Variable	**OR (95% CI)**	***P***	**OR (95% CI)**	***P***
Female (vs male)	0.878 (0.569–1.355)	0.557	0.661 (0.398–1.099)	0.110
Age p*er y*	1.030 (1.012–1.048)	0.001	1.031 (1.010–1.052)	0.004
Diabetes (vs no diabetes)	1.732 (0.996–3.011)	0.052	1.362 (0.737–2.516)	0.324
Prior CAD	1.546 (0.809–2.954)	0.187	–	
Chronic statins	1.179 (0.726–1.914)	0.507	–	
FH (DLCN score ≥ 6 vs ≤ 2)	1.384 (1.108–1.730)	0.004	1.248 (0.881–1.766)	0.212
LDL cholesterol. *Per mg/dL*	1.592 (1.113–2.278)	0.011	1.177 (0.672–2.059)	0.569
CRP ≥ 3 mg/L (vs CRP < 3 mg/L)	2.428 (1.559–3.782)	< 0.001	2.099 (1.326–3.323)	0.002

*OR* Odds ratio, *CI* confidence interval, *FH* familial hypercholesterolemia, *DLCN* Dutch Lipid Clinic Network, *LDL* Low density lipoprotein, *CAD* coronary artery disease, *CRP* C-Reactive Protein

## Discussion

**Fig. 4:**
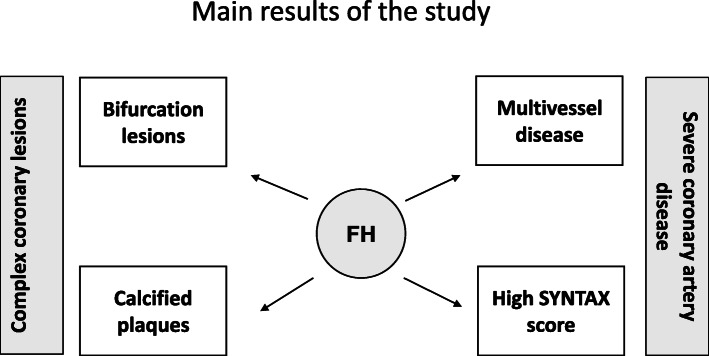
Cartoon representation of the main results

Only few studies have assessed the characteristics of coronary lesions in FH patients hospitalized for acute MI [7, 8, 12–15]. After matching for the main factors associated with CAD, the findings of this study suggest that the FH-associated high cholesterol burden, which starts at an early age, and inflammation are associated with CAD severity. Here, severe CAD is characterized by multivessel disease, a high SYNTAX score, and anatomical complexity features, including bifurcation lesions and calcified plaques (Fig. 4). These data are consistent with previous studies that included patients with genetically-determined FH [14, 16, 25].

Wang et al. [8] reported frequent multi-vessel lesions in FH patients, while non-FH patients had more frequent one-vessel CAD (multi-vessel CAD: 75.7% versus 34.1% and one-vessel CAD; 54.3% versus 21.6%, respectively, *P* < 0.001). This finding was also reported in 2 other studies, although in patients with possible FH [13, 14]. In a recent study of 382 young survivors (≤ 40 years old) of acute MI, patients with HF were three times more likely to have multiple vessel lesion location (36.2% versus 12.8%, *P* = 0.011) [26]. Similar to the current study, a small number of patients with angiographically healthy coronary arteries or with non-significant lesions were found, (*n* = 4), but these individuals were considerably less likely to be FH patients [12]. Two recent Chinese studies investigated CAD extension in FH patients [8, 15] using Gensini angiographic criteria [27], which is limited to severity of stenosis (estimated as a percentage), coronary plaque features and lesion location (proximal or distal). FH patients had more severe coronary injury [8, 15], and male sex was significantly associated with complex lesions, in agreement with previous studies [18, 24]. This work on a young FH population (mean age 51 years) further suggests that in addition to the LDL-C burden, inflammation plays a role in promoting the extension of CAD, as highlighted by higher CRP levels [28, 29].

To the best of our knowledge, this is the first study to use validated complexity criteria to evaluate the coronary lesions of FH subjects on coronary angiography [18, 24]. We found that the number of multiple complex lesions was mainly related to age and LDL-C levels. Moreover, bifurcated lesions, large calcifications, and the presence of multiple lesions were the key anatomical features characterizing complex CAD in FH patients. In asymptomatic FH patients, Pang et al. [16] also found more calcified plaques, especially on the left main artery, and a higher calcium score using coronary computed tomography (CT). PCI are high-risk procedures when done in calcified and bifurcated lesions, and recent studies, including a meta-analysis, have shown that these complex features have a major impact on the recurrence of ischemic events and long-term mortality [30, 31]. Moreover, in randomized clinical trials, the lesion complexity score was an independent predictor of short- and medium-term ischemic risk. The CHAMPION-PHOENIX trial, which included 10,854 patients with chronic or acute coronary syndrome, showed that a combined endpoint of all-cause death, recurrent MI, new revascularization guided by an ischemia test, or stent thrombosis within 48 h after PCI, was significantly related to the identified number of lesion complexity features (OR 1.68, 95% CI 1.20–2.36; OR 2.78, 95% CI 2.00–3.87; and OR 3.23, 95% CI 2.33–4.48, *P* < 0.0001, for 1, 2, and 3 complex features compared with no complex features, respectively) [24]. This association was observed up to 30 days of follow-up. In the DAPT study, patients with complex coronary anatomy (defined by the presence of at least 1 complexity criterion) had increased rates of major CV events (5.3% versus 3.5%; *P* < 0.001) and MI or stent thrombosis (3.9% versus 2.4%; *P* < 0.001) within 1 year, but these differences did not persist beyond 12 months [18]. Further work is needed to determine whether these characteristics could impact the short-term prognosis of FH patients after MI.

A recent French study on the 2005 and 2010 cohorts of the FAST-MI registry showed that an LDL-C target may be difficult to achieve in FH patients with acute MI. Even though they received intensive lipid-lowering therapy at discharge (statin + ezetimibe), FH patients had much higher LDL-C levels than non-FH patients at 5 years of follow-up (123 mg/dL and 83 mg/dL respectively, *P* < 0.001) [32]. In addition, and during intensive lipid-lowering treatment, FH patients had an increased risk of death, MI recurrence and stroke, even after adjustment for CV risk factors, suggesting the need for more aggressive management. On the other hand, and beyond LDL-C concentration, some factors such as female sex, high HDL-C levels, not smoking and elevated adiponectin may contribute to improved cardiovascular event-free survival in FH patients [33].

As secondary prevention, PCSK9 inhibitors such as alirocumab or evolocumab can be used to lower LDL-C and have demonstrated their clinical benefit in addition to intensive statin treatment [34]. Moreover, PCSK9 inhibitors provide better adherence than statins and can help to improve compliance to statin treatment in a real-world setting [35].. Among 4015 post-MI patients, it was demonstrated that full adherence to treatment is associated with a lower rate of adverse cardiovascular events after 2-years follow-up, and reduction of annual direct medical costs for MI hospitalization [36].

### Study strengths and limitations

The presence of DLCN criteria, such as tendon xanthomas or corneal arches, and a family history of high cholesterol or vascular disease were not collected in our database. This information bias may result in an underestimation of the true prevalence of FH. However, the FH probability rate found in our population (approximately 3%) is consistent with other major studies [9, 14, 25, 32]. In addition, it is likely that many of the FH patients in our study had tendon xanthoma. In 394 Japanese coronary patients undergoing PCI, most FH patients had Achilles heel xanthoma, which was predictive of the severity of coronary lesions [37]. Another recent series of 241 patients found that CAD patients had a high prevalence of Achilles heel xanthoma (18.2%), which was associated with multi-vessel coronary disease and imaging vulnerability criteria for atheromatous plaques [38]. Other missing data in our study include the statins doses, but we applied a correction factor of ≈30% to LDL-C levels in order not to overestimate the probability of FH. Moreover, genetic testing was not performed to confirm FH in the present study. In another recent study, a genetic diagnosis was obtained in 57 of 84 patients with DLCN ≥6 (67.9%) [39]. However, the procedure used to calculate the probability of FH with the adapted Dutch lipid Clinic criteria is widely used in routine clinical practice.

Finally, the retrospective design of the study may potentially bias the results.

## Conclusion

In patients with HF and acute MI, coronary lesions are anatomically complex, and characterized by multiple lesions, calcifications and bifurcation lesions. These features were associated with a high cholesterol burden and inflammation. The findings of this study reinforce the need for early screening for FH and highlight the fact that this condition is still under-treated. Aggressive cholesterol-lowering management is an important part of secondary prevention in these young high-risk patients.

## Data Availability

The data that support the findings of this study are available from Dijon-Bourgogne University Hospital. However, restrictions apply to the availability of these data, which were used under license for the current study and are thus not publicly available. Data can be made available from the authors upon reasonable request and with permission from the Dijon-Bourgogne University Hospital.

## Abbreviations

FH: Heterozygous familial hypercholesterolemia
LDL-C: LDL-cholesterol
PCSK9: Proprotein convertase subtilisin/kexin type 9
DLCN: Dutch Lipid Clinic Network
CV: Cardiovascular
CAD: Coronary artery disease
MI: Myocardial infarction
RICO: Côte d’Or Myocardial Infarction Observatory
PCI: Percutaneous coronary intervention
CABG: Coronary artery bypass graft surgery
LVEF: Left ventricular ejection fraction
HF: Heart failure
CT: Computed tomography
ARS: Agence Régionale de Santé
